# Stem Cells and Angiogenesis: Implications and Limitations in Enhancing Chronic Diabetic Foot Ulcer Healing

**DOI:** 10.3390/cells11152287

**Published:** 2022-07-25

**Authors:** Vikrant Rai, Rebecca Moellmer, Devendra K. Agrawal

**Affiliations:** 1 Department of Translational Research, Western University of Health Sciences, Pomona, CA 91766, USA; dagrawal@westernu.edu; 2 College of Podiatric Medicine, Western University of Health Sciences, Pomona, CA 91766, USA; rmoellmer@westernu.edu

**Keywords:** chronic inflammation, diabetic foot ulcers, neo-angiogenesis, stem cells, wound healing

## Abstract

Nonhealing diabetic foot ulcers (DFUs) are a continuing clinical issue despite the improved treatment with wound debridement, off-loading the ulcer, medication, wound dressings, and preventing infection by keeping the ulcer clean. Wound healing is associated with granulation tissue formation and angiogenesis favoring the wound to enter the resolution phase of healing followed by healing. However, chronic inflammation and reduced angiogenesis in a hyperglycemic environment impair the normal healing cascade and result in chronically non-healing diabetic foot ulcers. Promoting angiogenesis is associated with enhanced wound healing and using vascular endothelial growth factors has been proven beneficial to promote neo-angiogenesis. However, still, nonhealing DFUs persist with increased risks of amputation. Regenerative medicine is an evolving branch applicable in wound healing with the use of stem cells to promote angiogenesis. Various studies have reported promising results, but the associated limitations need in-depth research. This article focuses on summarizing and critically reviewing the published literature since 2021 on the use of stem cells to promote angiogenesis and enhance wound healing in chronic non-healing DFUs.

## 1. Introduction

The diabetic foot ulcer (DFU) is a vascular complication of diabetes mellitus (DM) affecting nearly 6.3% of the global population and a prevalence of 13% in the USA [1,2]. Wound debridement, off-loading the ulcer, medication, wound dressings, and preventing infection by keeping the ulcer clean are the gold standard in the treatment of diabetic foot ulcers (DFU). Despite the advancement in methods of wound healing, chronic DFU remains a critical clinical problem associated with costly and prolonged treatment, risk of amputation, and a high degree of morbidity and mortality. Diabetic vasculopathy, decreased angiogenesis, ischemia, chronic inflammation, and the wound environment interfering with the effect of endogenous factors regulating the healing response render the wound in a chronic inflammatory state without progressing to the resolution phase [3,4,5,6]. Wound healing comprises four phases including homeostasis/coagulation, inflammatory cell recruitment, the proliferative phase, and the maturation phase [6,7]. Angiogenesis and granulation tissue formation plays a critical role in wound healing by providing nutrition, oxygen, and matrix. Angiogenesis is increased in the early phase of healing while the density of blood vessels decreases with healing and scar formation. Granulation tissue acts as a matrix for proliferating vessels, fibroblast migration, and collagen formation. Impaired granulation tissue formation and angiogenesis are distinctive features of non-healing DFUs [8,9].

**Figure 1:**
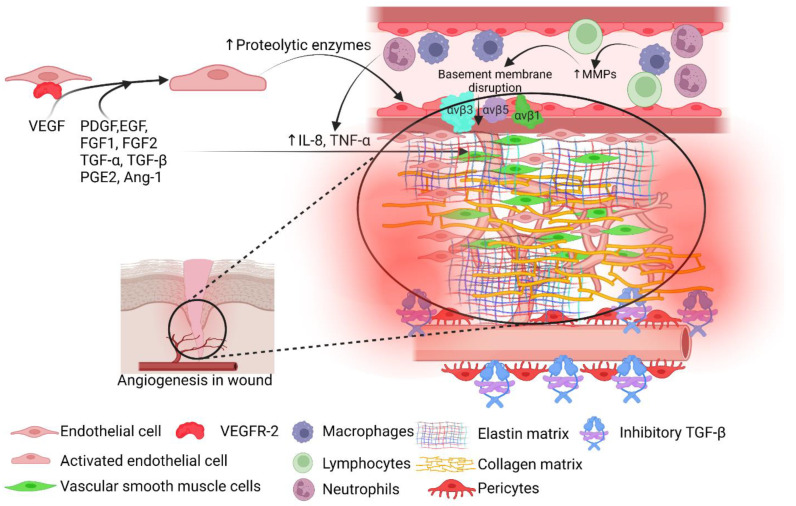
Angiogenesis in wound healing. Vascular endothelial growth factor (VEGF) secreted from fibroblasts in the wound environment activates endothelial cells (ECs) leading to increased secretion of proteolytic proteins. Increased matrix metalloproteinases (MMPs) from macrophages and proteolytic enzymes facilitate the disruption of the basement membrane, migration of ECs, and sprouting of the new vessels into the wound. This process is facilitated by increased expression of adhesion proteins (VCAM-1) and integrin receptors (αvβ 1, αvβ 3, and αvβ 5) and mediators of angiogenesis such as platelet-derived growth factor (PDGF), epidermal growth factor (EGF), fibroblasts growth factor (FGF), transforming growth factor (TGF)-α and β, prostaglandin E2 (PGE2), angiotensin (Ang)-1, interleukin (IL)-8, and tumor necrosis factor (TNF)- α. Increased recruitment of vascular smooth muscle cells (VSMCs) and pericytes facilitate neo-angiogenesis and vasculogenesis. Once vessels are formed and wound healing enters the later phase of healing angiogenesis is suppressed by the inhibitory form of TGF-β and increased secretion of endostatin (collagen XVIII).

The process of angiogenesis is well-orchestrated involving interaction between cellular and molecular mediators including fibroblast growth factor (FGF), vascular endothelial growth factor (VEGF), transforming growth factor (TGF)-β1, angiopoietin 2, and extracellular matrix (ECM) environment. Additionally, sprouty2, pigment epithelium-derived factor (PEDF), low-density lipoprotein receptor-related protein (LRP)6, thrombospondin (TSP)1, chemokine (C-X-C motif) ligand (CXCL)10, chemokine (C-X-C motif) receptor (CXCR)3, platelet-derived growth factor receptor beta (PDGFR-β), heparin-binding EGF-like growth factor (HB-EGF), epidermal growth factor receptor (EGFR), semaphorin3a, neuropilin 1, neural/glial antigen (NG)2, laminin 8, laminin 10, and regulator of G protein signaling (RGS)5 also regulate angiogenesis during wound healing. ECM intricately regulates angiogenesis and vasculogenesis by regulating the expression of the ECM receptor, αvβ3, the integrin receptor for fibrin and fibronectin [9,10,11,12]. Cytokines secreted from infiltrating macrophages and fibroblasts support ECM formation and angiogenesis. The expression of integrin receptors mobilizes the sprouting vessels to penetrate fibrin and fibronectin-rich tissue aiding in wound healing. However, when granulation tissue and ECM are replaced by collagen-rich tissues, vessel formation regresses and degenerates through apoptosis involving anti-angiogenic factors [11] (Figure 1).

Therapeutic strategies involving growth factors (PDGF, EGF, FGF, and VEGF), non-growth factor proteins (insulin, erythropoietin, stromal-cell derived factor-1, spidroin, and thymosin beta 4), peptides (anti-microbial peptides, cathelicidins, LL-37 derivatives, and vasoactive intestinal peptide), blood-derived factors (PDGF-BB, TGF-β1, FGF-2, VEGF-A, EGF, and platelet factor-4), microRNAs, drugs and small molecules (adenosine triphosphate, statins, deferoxamine, natural compounds, and hyaluronan), nanomaterials in the scaffold, and stem cells (adipose-derived stem cells, bone marrow-derived mesenchymal stem cells, induced pluripotent stem cells, and placenta-derived mesenchymal stem cells) have been discussed to enhance angiogenesis and wound healing [13,14]. Becaplermin, a recombinant platelet-derived growth factor, is an FDA-approved drug to treat a neuropathic ulcer in diabetes, systemic bioavailability is a limitation [15]. Thus, exploring new treatment strategies is warranted for the treatment of ischemic DFUs. Using stem cells for wound healing is another promising approach. Multiple articles in the literature have reviewed the mechanism and the role of stem cells in enhancing wound healing [16,17,18,19,20,21,22,23]. Hence, this review focuses on the role of stem cells in promoting angiogenesis to enhance wound healing.

## 2. Angiogenesis and Wound Healing

**Figure 2:**
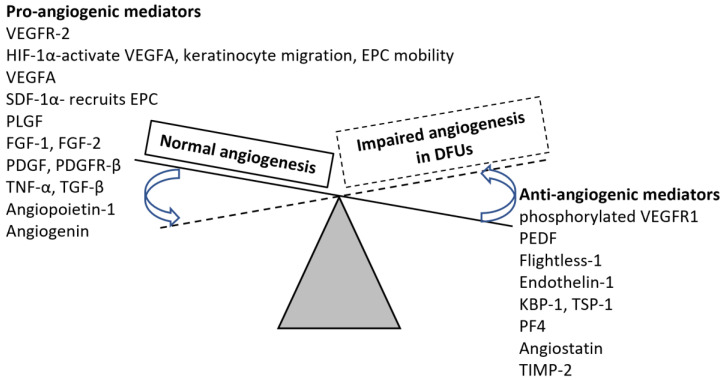
Change in the expression levels of pro-and anti-angiogenic factors in chronic nonhealing diabetes foot ulcers. Normal angiogenesis occurs with increased expression of proangiogenic and decreased expression of antiangiogenic mediators. The expression of proangiogenic factors decreases while antiangiogenic mediators increase in nonhealing diabetic foot ulcers. Vascular endothelial growth factor receptor (VEGFR)-2, hypoxia-inducible factor 1-alpha (HIF-1α), vascular endothelial growth factor (VEGF)-A, stromal cell-derived factor (SDF)-1α, placental growth factor (PLGF)—a member of the VEGF family, fibroblast growth factor (FGF), platelet-derived growth factor (PDGF), platelet-derived growth factor receptor (PDGFR)-β, tumor necrosis factor (TNF)-α, transforming growth factor (TGF)-β, pigment epithelium-derived factor (PEDF), Krüppel binding protein (KBP)-1, thrombospondin (TSP)-1, platelet factor (PF)-4, and tissue inhibitors of matrix metalloproteinases (TIMPs). The continuous line shows the expression levels during normal wound healing while the dotted line and arrows represent the shift in expression levels.

The process of angiogenesis is well regulated by angiogenic (TGF-β, TNF-α, VEGF, PDGF, FGF, angiogenin, and angiopoietin-1) and anti-angiogenic (angiostatin, TIMP-2, TSP-1, endostatin, sprouty 2, PEDF, PF-4, IFNα/β) factors [8,12,24] involving oxidative stress (HIF-1α), inflammation, and Sonic Hedgehog (Shh) signaling. Reduction of angiogenesis in the last phase of wound healing is essential for proper wound healing without scarring and selective reduction of angiogenesis and inflammation has been suggested for proper wound healing [12]. This suggests that increased angiogenesis plays a critical role in normal wound healing during the early phase, but attenuation of angiogenesis is needed for proper healing. However, this dynamic regulation of angiogenesis may alter during diabetes due to changes in the expression levels of pro-and anti-angiogenesis proteins (Figure 2), however, the available literature is not conclusive [25]. The inconsistencies between the results of the expression of pro-and anti-angiogenic factors may be due to loss of tissue, presence of necrosis and gangrene, the differential response of fibroblasts to hypoxia, different levels of inflammation, tissue fibrosis, vascular thrombosis, arteriolar sclerosis, differential expression of these mediators in different cells in healthy versus wound tissues and healthy versus diabetic tissue as well as different levels of hyperglycemia. Another issue may be a differential expression between different types of diabetic ulcers. Difficulties in translating the lab findings to therapy in clinics to improve angiogenesis and wound healing are also due to the differential expression of these mediators in human versus murine models [25]. Temporary hypoxia is needed for angiogenesis as increased expression of HIF-1α dimerizes and activates hypoxia response elements causing increased expression of VEGF, but the presence of hyperglycemia affects the stability and activation of HIF-1α and attenuates angiogenesis through suppression of PDGF, VEGF, and TGF-β [26].

## 3. Stem Cells and Angiogenesis in DFUs

Angiogenic therapy using stem cells to enhance healing in refractory wounds has shown promising therapeutic efficacy. Stem cells migrate to the site of the wound, differentiate, proliferate, promote granulation tissue formation, collagen deposition, and angiogenesis, and ameliorate neuro-ischemia and inflammation thereby enhancing wound healing. Angiogenesis is promoted by increasing the secretion of angiogenic factors including VEGF and Von Willebrand factor and endothelial cell recruitment through TNF- α. Mesenchymal stem cells (MSCs) have been used to promote angiogenesis in pre-clinical and clinical studies [16,18,27]. Paracrine signaling and the capability of stem cells to differentiate into specialized cells including fibroblasts, vascular endothelial cells, and epithelial cells contribute to the efficacy of stem cells in stimulating angiogenesis, neovascularization, and re-epithelialization. Stem cells heal the wound by providing a favorable environment by secreting cytokines, chemokines, and growth factors necessary to produce an extracellular matrix and promoting neo-angiogenesis. Thus, stem cells alter the wound microenvironment favorable for healing and promote tissue regeneration at the wound site. Administration of stem cells is associated with the advantage of angiogenesis promotion, attenuated inflammation, and enhanced wound healing but is also associated with side effects (Table 1). Further, stem cells have the advantage of administering them along with other treatments to exploit their role in a better way for the treatment of nonhealing DFUs [16,17,18,28].

Mesenchymal stem cells induce the mobilization of various angiogenesis factors including SDF-1, VEGF, EGF, insulin-like growth factor-1 (IGF-1), angiopoietin (Ang)-1, keratinocyte growth factor (KGF), MMP-9, macrophage inflammatory protein (MIP)-1α and β and erythropoietin (EPO) to the wound bed stimulating recruitment, proliferation, and differentiation of endothelial progenitor cells and thereby angiogenesis and wound healing [16,27]. Among the MSCs, bone marrow-derived MSCs are more suitable to enhance healing in DFUs [17]. MSCs have been used in various studies and shown to have a beneficial role in enhancing wound healing in DFUs. The molecular mechanism involved in enhancing wound healing in DFUs using stem cells has been extensively reviewed [17,29]. Adult stem cells including bone marrow-derived mesenchymal stem cells (BM-MSC), peripheral blood-derived mesenchymal stem cells (PB-MSC), human umbilical cord-derived mesenchymal stem cells (hUC-MSC), and adipose-derived mesenchymal stem cells (ADSC) have been used in pre-clinical and clinical studies (Table 2). Additionally, enhanced wound healing in a murine model of DFUs using human amniotic MSCs, the micronized amniotic membrane containing human amniotic epithelial cells, human placental MSC, collagen gels containing embryonic fetal liver MSCs, and collagen hydrogel scaffold to deliver human fetal aortic MSCs has also been reported. Moreover, the use of embryonic stem cells, induced pluripotent stem cells, and granulocyte-colony stimulating factors stimulating bone marrow to mobilize endothelial progenitor cells at the wound site, have also been documented and reviewed [19,20,21,22,23].

Regarding the route of administration of stem cells to enhance wound healing, nonvascular administration including intradermal, subcutaneous, and intramuscular injections are the most used routes to enhance DFU wound healing. Additionally, local administration of stem cells in a collagen-based hydrogel and systemic venous and arterial administration of stem cells have also shown promising results [19,20]. Further, Yan et al. [37] reported that both systemic and topical application of BM-MSCs have the potential of enhancing wound healing and promoting neo-angiogenesis and vascularization but systemic administration is associated with ameliorating hyperglycemia and improving blood perfusion of the ischemic hindlimb of the diabetic rats with a positive wound distribution and trans-differentiation to ECs.

## 4. Stem-Cell Derived Exosomes and Combinational Strategies to Enhance Diabetic Wound Healing

Stem cells not only enhance angiogenesis but also attenuate the effects of diabetes and protect endothelial cells [36]. Low cell retention and integration of stem cells are issues of using hydrogels while administering stem cells to enhance wound healing in DFUs. Shi et al. [38] reported that gelatin microspheres enhance the delivery and integration of locally delivered adipose-derived stem cells from rats. The use of gelatin microspheres was associated with M2 macrophage polarization, collagen deposition, angiogenesis, peripheral nerve recovery, and hair follicle formation suggesting the efficacy of using microspheres in enhancing healing in DFUs. In another study, Takahashi et al. [39] reported that topical application of hydrogels containing conditioned medium from hypoxically cultured amnion-derived mesenchymal stem cells promotes wound healing in diabetic mice by enhancing angiogenesis, accelerating epithelization, and suppressing inflammation. The result of this study obviates the need for stem cell transplantation at the wound site though future research is warranted.

Along with transplanting stem cells in gels or as nanoparticles, exosomes derived from stem cells have also shown a beneficial effect in enhancing angiogenesis and wound healing in DFUs. A study by Wang et al. reported the effectiveness of exosomes derived from epidermal stem cells in improving diabetic wound healing [40]. The study reported that compared to epidermal stem cells (ASC) alone, exosomes isolated from ASC (ASC-Ex) showed better results in wound healing in db/db mice. Enhanced wound healing with ASC-Ex was associated with decreased inflammation, augmented wound cell proliferation, stimulating angiogenesis, and inducing M2 macrophage polarization. Heras et al. [41] reported increased angiogenesis and human dermal fibroblasts proliferation and migration, in vivo, with exosomes isolated from hair follicle-derived MSCs (HF-MSCs) and adipose tissue-derived MSCs (AT-MSCs). Another study by Gondaliya et al. reported enhanced collagen deposition, angiogenesis, and re-epithelialization in diabetic wounds with MSC-derived exosomes loaded with miR-155 inhibitor in a mouse model [42]. Accelerated wound healing was associated with keratinocyte migration, restoration of FGF-7 levels, and decreased inflammation. The promotion of angiogenesis with accelerated diabetic wound healing in diabetic mice was also reported with exosomes derived from human umbilical cord mesenchymal stem cells via oxidative stress amelioration [43]. Decreased angiogenesis or neovascularization in hyperglycemic state and old age may be due to senescent endothelial cells (ECs) or decreased ECs proliferation. Xiao et al. [44] reported that mesenchymal stem cell-derived small extracellular vesicles (MSC-sEV) mitigate oxidative stress-induced ECs senescence and stimulate angiogenesis through miR-146a/Src. Increased angiogenesis to enhance wound healing in diabetic rats by promoting HIF-1α-mediated angiogenesis in the PI3K-AKT-mTOR dependent manner with extracellular vesicles derived from human adipose-derived stem cells (hADSC-EVs) in association with accelerated wound healing was reported by Liu et al. [45]. These findings suggest that mitigating EC senescence may enhance angiogenesis and thus might be of therapeutic significance to enhance healing in chronic non-healing DFUs. Further, stem cells have better therapeutic effects in combinations with hydrogels, collagen matrix, and nanoparticles in comparison to stem cells alone and pretreatment will enhance efficacy (Table 3).

Many studies have also reported enhanced therapeutic efficacy of adipose stem cell released extracellular vesicles and exosomes are being used to enhance wound healing in DFUs [61,62,63]; it is imperative to further investigate the strategies to enhance the therapeutic efficacy of stem cells and their synergistic or additive effects in a combinational approach to promote angiogenesis and wound healing in chronic non-healing DFUs. In-depth research involving exosomes (EVs) isolated from stem cells is also important because EVs isolated from different types of stem cells may have differential effects on angiogenesis and wound healing as reported by Pomatto et al. [64] that EVs isolated from bone marrow and adipose mesenchymal stem cells have differential therapeutic effects on wound healing and angiogenesis. Although these studies (Table 2 and Table 3) suggest the promising role of stem cells in inducing angiogenesis in the presence of hyperglycemia most of the studies conducted on the murine model were done either on streptozotocin-induced diabetes mellitus, type I diabetes, or genetically induced diabetic mice. However, type II diabetes is common in human patients with DFUs. Having said that, it is warranted to design clinical trials to establish the role of stem cells in enhancing angiogenesis in DFU wound healing.

## 5. Limitations and Future Perspectives

Limited cell viability, reduced cell number, limited proliferation, and differentiating capacity of the stem cells are limiting factors while using stem cells. Stem cells may be autologous or allogenic. Both autologous and allogeneic stem cells have shown improved wound healing in human and animal models, but the pre-clinical and clinical data is still limited and more well-planned studies with an increased number of subjects are warranted for the feasibility of using stem cells in enhancing healing in DFUs [17]. Decreased viability of stem cells due to late glycosylation end products in a hyperglycemic environment diminishes the efficacy of stem cells in enhancing wound healing in DFUs [65]. However, a study by Assis et al. [66] reported that the MSCs from diabetic patients have the same potential for angiogenesis if they are seeded on decellularized micro fragments. The MSCs were isolated from the bone marrow of distal tibial or femoral shafts from the amputated limb. These results suggest the efficacy of autologous SMCs even in a diabetic patient in enhancing angiogenesis during wound healing.

Administering an increased number of stem cells might be an alternative but is associated with over-proliferation and tumorigenicity [67]. Administering stem cells with a biomaterial carrier may mitigate the limitations associated with the number and viability of stem cells but needs extensive research [19,20]. Administration of preconditioned stem cells with photo-biomodulation is another strategy to enhance the viability and activity of stem cells to enhance angiogenesis and wound healing [68,69,70]. With the background of the complications associated with autologous MSCs, Palma et al. [71] reported that one specific subpopulation derived from the Wharton’s jelly of the umbilical cord (ucMSC), the differentiated mesenchymal cells (DMCs), has the potential of improving wound healing with increased angiogenesis without any rejection of the transplanted cells in immunocompetent mice. These results showed successful allogeneic transplantation of MSCs. Treating chronic diabetic foot ulcers with adipose-derived stromal vascular fraction cell injections may be a safe strategy to induce vascular repair, angiogenesis, and wound repair [72]. Although the studies discussed in this article showed promising results favoring angiogenesis and wound healing and the limitations of stem cells, the heterogeneity among the results of various randomized clinical trials in human patients also warrants well-planned large-scale randomized clinical trials to establish the best protocols, doses, transplant type (autologous, allogenic, xenotransplant), cell types, cell source, and administration routes to enhance angiogenesis and wound healing [19,20].

## 6. Conclusions

Stem cells have been proven beneficial in promoting angiogenesis and enhancing wound healing both in vitro and in vivo but most of the in vivo studies were conducted using animal models including mice, rats, and porcine. The results of various studies showed heterogeneous results due to different sources of stem cells, route of delivery, and stem cell properties like viability and proliferation after administration. Further, EVs isolated from stem cells and a combination of stem cells with other strategies, as discussed here, has also shown promising results. Based on this background, large-scale clinical trials are warranted to harness the beneficial outcome of the lab findings and translate them to the clinics.

## Figures and Tables

**Table 1:** Advantages and limitations of the stem cells enhancing healing in DFUs. Stem cells showed improved clinical efficacy in enhancing wound healing in DFUs by increased angiogenesis and re-epithelialization [17]. The common side effects associated with stem cell therapy are whole body urticaria, diarrhea, oral ulceration, the elevation of serum creatinine level, number, and differentiated potential decline with aging, nausea, and vomiting. Bone marrow-mesenchymal stem cells (BM-MSCs), umbilical cord blood-derived mesenchymal stem cells (UCB-MSCs), adipose-derived mesenchymal stem cells (AMSCs), umbilical cord-derived mesenchymal stem cells (UC-MSCs), placenta-derived mesenchymal stem cells (PMSCs), and human amniotic fluid-derived stem cells (AF-MSCs), human gingiva-derived mesenchymal stem cells (GMSCs).

Source of Stem Cells	Advantages
BM-MSCs	No immunologic restriction, does not stimulate alloreactivity, escape lysis by T-cells and NK cells Reduced formation of cytotoxic lymphocytes suppresses T-cell-derived IFN-γ, intramuscular transplantation
UCB-MSCs	Similar morphology and cell surface antigens, the potential of differentiation into BM-MSCs Short doubling time, long viable time, anti-inflammatory activity, intramuscular transplantation
AMSCs	Characteristics like BM-MSCs, subcutaneous injection
UC-MSCs	Rich, safe, of short doubling time, and easy to collect Fibroblastic morphology, typical immunophenotypic markers, and multiple differentiation potential to BM-MSCs, lower immunogenicity
PMSCs	A large number of cells can be isolated, better proliferation capacity, intraperitoneal administration The morphology, size, surface phenotype, and immunosuppressive characteristics are like BM-MSCs
AF-MSCs	A large number of cells can be isolated from the small volume, remain stable, have a high proliferative capacity, multilineage differentiation potential, immunomodulatory activity Lack of significant immunogenicity
GMSCs	Homogenous, not tumorigenic, easy to isolate, stable phenotype, can be isolated without ethical problems, greater capacity of proliferation and migration than AMSCs and BM-MSCs without growth factors

**Table 2:** Stem cells enhance angiogenesis and wound healing in DFUs. Neonatal porcine bone marrow-derived mesenchymal stem cell (npBM-MSC), Adipose-derived mesenchymal stem cells (ADSCs), human induced pluripotent stem cell-derived smooth muscle cells (hiPSCSMC), endothelial differentiated adipose-derived stem cells (EC-ADSCs), human umbilical cord blood-derived CD34^+^ stem cells (UCB-CD34^+^SC), bone marrow mononuclear cells (BMMNCs), endothelial progenitor cells, human fetal aorta-derived CD133^+^ progenitor cells and their conditioned medium (CD133^+^ CCM), human umbilical cord-derived MSCs (hucMSCs).

Stem Cell	Strategy	Parameters Checked	Outcome
npBM-MSC [29]	Xenotransplantation in mice model of diabetic wound.	Rate of wound closure and the promotion of neovascularization	The wound closure rate was significantly improved on postoperative days 4 and 7 Promoted angiogenesis and lymphangiogenesis
ADSCs [30]	Autologous transplantation in mice	Lymphangiogenesis Wound healing	ADSCs accelerate lymphatic endothelial cells proliferation, migration, and lymphangiogenesis ADSCs enhance VEGFR3-mediated lymphangiogenesis via METTL3-mediated VEGF-C m6A modification to improve wound healing in DFUs
hiPSCSMC [31]	Xenotransplantation hiPSC-SMC embedded in 3D collagen scaffolds were applied to diabetic, nude mice with splinted back wounds	To compare angiogenic factor secretion from ADMCs and hiPSCSMC	hiPSC-SMC secretes increased concentration of pro-angiogenic cytokines compared with murine ADMCs. hiPSC-SMC-containing collagen scaffolds accelerate diabetic wound healing hiPSC-SMC increases the number of total and M2 macrophages hiPSC-SMC increases angiogenesis via VEGF-A and TGF-β1
ADSCs [32]	Autologous transplantation in swine	Percentage of wound healing	Increased percentage of wound closure rates with ADSCs and EC-ADSCs, and conditioned media Increased angiogenesis with stem cell therapy Significant decrease in inflammation with stem cells
hUCB-CD34+SC [33]	Xenotransplantation in a rat model of diabetic wound locally.	To evaluate the efficacy of stem cells in the healing of wounds	A significant decrease in mean wound surface area, increase in mean epidermal thickness, blood vessel proliferation, and collagen deposition
EPCs-BMMNCs	Autologous stem cells transplantation in mice topically	Wound healing and angiogenesis	Accelerated wound healing and induced expression of VEGF and bFGF promoting angiogenesis
CD133+ CCM [34]	Xenotransplantation in mice model of diabetic wound	Wound healing and angiogenesis	Stimulation of migration, angiogenesis-like network formation and induction of Wnt expression Stimulate wound healing by paracrine mechanisms
ABCB5+MSCs [35]	Human dermal ABCB5+ MSCs were transplanted via intramuscular injection in mice ischemic limb and topically in human DFUs	To evaluate the angiogenic potential of ABCB5+ MSCs	In mice Accelerated perfusion recovery of ischemia Increased angiogenesis Clinical trial in human Reduction in wound surface area in therapy refractory DFUs with topical application
hucMSCs [36]	hucMSCs were infused in diabetic rat	Repair of diabetic vascular endothelial cell damage	hucMSCs ameliorated blood glucose and protected vascular endothelium from diabetic damage through paracrine effect involving MAPK/ERK signaling

**Table 3:** Combinational strategies with stem cells enhancing angiogenesis and wound healing in DFUs: Exendin-4 (Ex-4) is a glucagon-like peptide-1 receptor agonist known to have many beneficial effects on diabetes, human adipose tissue-derive stem cells (hADSCs), long noncoding RNA (Lnc), bone marrow mesenchymal stem cells (BMSCs), self-assembled nano-peptide hydrogels with human umbilical cord mesenchymal stem cell spheroids (hUC-MSCsp), acellular dermal matrix (ADM), extra cellular matrix (ECM), hBM-MSCs/T/H/S embedded in an ECM scaffold (S) and preconditioned with hypoxia (H) and the β-adrenergic receptor antagonist, timolol (T), Wharton’s jelly mesenchymal stem cell (WJMSC), sodium ascorbyl phosphate (SAP), platelet rich plasma (PRP), human umbilical cord mesenchymal stem cells (hUC-MSCs), 5-aminolevulinic acid photodynamic therapy (ALA-PDT), EVs secreted by human umbilical cord mesenchymal stem cells (hucMSC-EVs), HOTAIR-MSC EVs- extracellular vesicles (EVs) isolated from mesenchymal stem/stromal cells (MSCs) transfected to overexpress long non-coding RNA HOX transcript antisense RNA (HOTAIR), endothelial cells (ECs), catechol-functionalized hyaluronic acid (HA-CA) patch.

Treatment	Combination/Strategy	Assessing Parameters	Study Outcome
Exosomes from linc00511-overexpressing ADSCs [46]	hADSCs-derived exosomes were injected into Sprague–Dawley (SD) rats along with human blood-derived EPC	Angiogenesis and wound healing Underlying molecular mechanism	Accelerate angiogenesis and wound healing by suppressing PAQR3-induced Twist1degradation
BMSC-derived exosomal lncRNA KLF3-AS1 [47]	Exosomes were delivered via tail vein injection in diabetic BALB/C mice	Wound healing Angiogenesis	Induction of angiogenesis to promote diabetic cutaneous wound healing.
Exosomes from pioglitazone pretreated MSCs [48]	Exosomes isolated from supernatants of pioglitazone-treated BMSCs (PGZ-Ex) were injected around the wounds by multisite subcutaneous injection	Wound healing Angiogenesis	PGZ-EX accelerates diabetic wound healing via enhanced angiogenesis, increased collagen deposition, ECM remodeling, and increased VEGF and CD31 expression
hucMSC-EVs [49]	hucMSC-EVs applied locally to diabetic mice	Angiogenesis Wound healing	hucMSC-EVs have regenerative and protective effects on high glucose-induced endothelial cells involving the transfer of miR-17-5p to target PTEN/AKT/HIF-1α/VEGF pathway hucMSC-EVs promote angiogenesis and accelerate wound healing
HOTAIR-MSC EVs [50]	HOTAIR-MSC EVs were injected around the wound in Sprague–Dawley rats	Wound healing Angiogenesis	HOTAIR-MSC EVs promote angiogenesis and wound healing in diabetic (db/db) mice.
Exendin-4 with ADSCs [51]	hADSCs were injected intradermally around the wound in db/db mice and Ex-4 was applied topically	Wound size Wound histology Angiogenesis	The combination of topical treatment of Ex-4 and injection of ADSCs has a better effect therapeutically than Ex-4 alone
hUC-MSCsp [52]	hUC-MSCsp transplanted into wounded skin of mice model of diabetes	Wound healing Angiogenesis Inflammation Comparison between stem cells alone and in combination	Accelerated wound healing Inhibited inflammation Promotes angiogenesis
ADSCs [53]	ADSCs in the acellular dermal matrix under hypoxic and normoxic conditions applied over DFU in a diabetic rat	Stem cell viability under hypoxic and normoxic conditions	The transplanted cells in the hypoxic-ADSCs/ADM membrane can survive longer at the chronic ulcer site and enhance angiogenesis, inhibits inflammation, and increase ECM formation
hBM-MSCs [54]	hBM-MSCs/T/H/S administered to porcine wound model	Wound healing Angiogenesis	MSC/T/H/S promoted wound re-epithelialization and angiogenesis and improved wound healing
WJMSC [55]	WJMSC with PF-127 hydrogel and SAP were transplanted onto excisional cutaneous wound bed in type II diabetic Sprague–Dawley rats	Wound healing Mitochondrial damage and oxidative stress	Promoted diabetic wound healing Decreased M1 and increased M2 macrophages Increased angiogenesis
ADSCs [56]	ADSCs (isolated from rats) alone and ADSCs with PRP was injected at the wound base and edges of diabetic Albino rats	To compare the efficacy of ADSC alone vs. ADSC+ PRP in wound healing and angiogenesis	PRP+ADSCs compared to their individual use are better for re-epithelialization, granulation tissue formation, collagen deposition, epidermal thickness, and angiogenesis by modulating the Notch pathway
ADSCs [57]	ADSCs (isolated from rats) combined with PRP were injected to wound in Sprague–Dawley rats	Angiogenesis Wound healing	ADSCs-PRP induced a higher wound closure rate Increases the expression of VEGF, p-STAT3, and SDF-1 Promote ECs proliferation thereby neovascularization
hUC-MSCs [58]	hUC-MSCs combined with ALA-PDT- hUC-MSCs were injected intradermally to diabetic C57BL/6J mice after exposing the mice to ALA-PDT with 10% ALA gel and 25 J/cm^2^ of PDT.	To investigate the efficacy of the combinational approach on wound closure, angiogenesis, and inflammation	Combining ALA-PDT with hUC-MSCs possesses a significantly enhanced therapeutic efficacy in enhancing wound healing, promoting angiogenesis, and attenuating inflammation and bacterial load suggesting its efficacy in healing refractory wounds.
ADSCs [59]	ADSCs combined with HA-CA ADSCs were injected around the wound in diabetic C57BL/6 mice and a patch was deposited on the wound	Angiogenesis Wound healing	HA-CA + ADSCs enhanced wound healing and angiogenesis synergistically involving PI3K/AKT pathway.
ADSCs [60]	Human ADSCs with SDF-1α gene-activated scaffold were tested in vitro using HUVEC	Pro-angiogenic properties	SDF-1α gene-activated scaffold overcomes the deficiencies associated with diabetic ADSCs and restores pro-angiogenic features ln ADSCs

## Data Availability

Not applicable.
